# A comprehensive review on learning curve associated problems in endoscopic vein harvesting and the requirement for a standardised training programme

**DOI:** 10.1186/s13019-016-0442-y

**Published:** 2016-04-08

**Authors:** Bhuvaneswari Krishnamoorthy, William R. Critchley, Rajamiyer V. Venkateswaran, James Barnard, Ann Caress, James E. Fildes, Nizar Yonan

**Affiliations:** Department of Cardiothoracic Surgery, University Hospital of South Manchester NHS Foundation Trust, Manchester, M23 9LT UK; The Transplant Centre, University Hospital of South Manchester NHS Foundation Trust, Manchester, M23 9LT UK; Institute of Inflammation and Repair, Faculty of Medical and Human Sciences, University of Manchester, Manchester, M13 9PL UK; The School of Nursing, Midwifery and Social Work, University of Manchester, Manchester, M13 9NT UK

**Keywords:** Coronary artery bypass, Saphenous vein, Endoscopic vein harvesting, Learning curve

## Abstract

Endoscopic vein harvesting is becoming one of the most favourable vein harvesting techniques in multiple bypass coronary surgery, due to its short term post-operative benefits with high patient satisfaction. However, long-term graft patency has been both supported and questioned in the literature. Graft failure can be affected by harvesting methods and operator’s experience. Endoscopic vein harvesting is associated with a learning curve period, during which the incidence of vein trauma is high due to unfamiliarity with the surgical technique. There is a paucity of structured learning tools for novice practitioners, meaning that training differs significantly between hospital centres. Inconsistent training methods can lead to poor surgical technique, which can have a significant impact on vein quality and stress level of the practitioner. In turn, this can lead to increased postoperative complications and longer surgical duration. The main aim of this literature review is to understand the impact of the learning curve on the vein conduit and whether there is a requirement for a standardised training programme for the novice practitioners.

## Background

Coronary artery bypass grafting (CABG) is one of the most common cardiac surgical procedures performed worldwide [1]. Despite arterial conduits having a superior long-term graft patency rate, the long saphenous vein is still the first choice conduit as a second graft in multi-vessel bypass grafts [2, 3]. Endoscopic vein harvesting (EVH) has become one of the most favourable techniques for conduit retrieval due to the reduction in wound complications, ameliorated postoperative pain and improved cosmetic outcome compared to traditional harvesting methods. However, no consensus has been reached regarding long term graft patency, with both positive [1, 4, 5] and negative [6, 7] data reported in clinical [8, 9] and histological studies [10]. A major impediment to long term bypass success is vein graft failure or occlusion, which can occur early or late. Numerous factors contribute to vein graft failure, including conduit quality [11, 12], graft diameter [13], type of graft [14, 15], grafting site [16], handling of the conduit [17], surgical conduit preparation [18], grafting technique [16, 17], patient risk factors [19] and technical error [17, 20]. Recent evidence also suggests that the harvesting method used [8, 21] and operator ability/experience [6] are of vital importance. This literature review seeks to address the effect of the EVH learning curve period on patient safety and highlights potential methods to minimise the impact of practitioner inexperience.

## EVH safety: Current evidence

Lopes et al. reported significantly inferior clinical outcomes in patients receiving conduits obtained by EVH compared to traditional harvesting [7]. This finding prompted a shift away from the use of EVH in many centres throughout Europe. However, a number of additional studies have disagreed with these results. A randomised study comparing EVH vs OVH by Yun et al. recruited *n* = 200 patients (*n* = 100 in each group) to assess graft patency and wound infections at 6 months [8]. They reported that EVH was associated with reduced risk of leg wound infection compared with OVH (7.4 % vs 19.4 %; *p* = 0.014) and the risk of graft failure was not significantly different (21.7 % EVH vs 17.6 % OVH, *p* = 0.584). Similarly, Allen et al. conducted a randomised trial of 112 patients and reported no significant differences over 5 years, including recurrent angina, myocardial infarction and death (EVH 75 % vs OVH 74 %; *p* = 0.85) [9]. More importantly, a cohort study comparing 8542 patients over 4 years reported that patients undergoing EVH had a lower mortality than OVH patients (11.3 % for EVH versus 13.8 % for OVH; *p* < 0.001) [4]. A more recent systematic review with meta-analysis including 27,789 patients concludes that EVH minimises the incidence of leg wound infections without increasing the mid-term risk for vein graft failure, myocardial infarction and mortality [22]. However, there is a huge gap within the literature regarding current methods of EVH training and their associated strengths and weaknesses. Furthermore, it is clear from the literature that there is no evidence of a structured training programme implemented for EVH that is comparable to that used as standard in other surgical specialities.

## EVH learning curve-related complications

Whilst the current evidence suggests that EVH is a safe procedure, it is apparent from a recent meta-analysis that novice practitioners may cause greater trauma to the vein compared to their experienced counterparts [22]. Whilst this meta-analysis discussed the potential impact of the learning curve, there was no mention of the impact of different training methods or the need to develop a standardised protocol for teaching novice practitioners. The current published evidence discusses the impact of EVH, its associated concerns, technical difficulties and poor conduit quality. However, as yet there is a lack of understanding as to how to rectify these problems, improve the surgical experience and minimise learning curve associated problems for the novice practitioners. A number of quantitative measures of vein quality have provided substantial evidence that operator ability and level of experience significantly impacts on the quality of the vein graft. As conduit quality directly affects graft patency and long term clinical outcome after CABG surgery, the implications of this are significant [6, 23]. Indeed, the significant effect of learning curve associated problems for EVH has led many to question the technique as a whole, despite its well-established post-operative benefits in the short term.

## Conduit repairs

The number of conduit repairs required following harvesting has been demonstrated to be inversely proportional to the level of experience accrued by the practitioner [24]. This is due to the nature of the endoscopic technique, which requires more direct manipulation and handling of the vessel than traditional open vein harvesting methods [25].

## Greater conversion rate from EVH to OVH during the learning period

The current literature suggests that the rate of conversion from an EVH to OVH procedure during the learning curve period range from 3 to 15 % [26]. This conversion rate is higher than expected from experienced practitioners due to the unfamiliarity with the technique [20, 27]. During the learning curve period, the chances of haematoma formation are significantly increased and conversion to the open technique necessitates severe trauma to the tissues which predispose to leg wound infections and also more frequent visits to postoperative clinics [28]. However, the cosmetic results of EVH are excellent and there is a significant reduction in immediate postoperative complications once the practitioner overcomes the learning curve [20, 27, 28]. Additionally, the current literature also suggests that practitioners with 100 or more cases of EVH experience have shorter harvesting times with improved conduit quality [29].

## Graft failure

Vein conduits undergo many changes once grafted into the arterial circulation [18]. A failure rate of 10–20 % is observed within a year [30], an additional 5–10 % within 5 years and at 10 years almost 50 % of conduits are blocked due to progressive disease [18]. There are many reasons for graft progressive atherosclerosis [31] and poor patency rate, such as graft spasm, sub-standard grafting techniques and thrombosis [18]. One of the major reasons for poor graft patency is progressive neointimal hyperplasia which is influenced by patient selection, relevant comorbidities [32], surgical technique, method of harvesting and the intrinsic quality of the conduit [11].

## Anxiety

Learning endoscopy in the clinical theatre setting promotes learner anxiety and exposes the patients to the risk of procedure-related education [33]. Furthermore, this approach results in variable learning experiences [34]. This may lead to an increase in the number of unnecessary complications such as trauma to the vein occurring due to technical error [5]. The time, number of patients and expense spent acquiring basic endoscopic skills in the operating room must also be considered [35]. A theoretical-based, evidence-supported surgical training tool is necessary to reduce EVH trainee anxiety around real patients, and would allow proper monitoring of technical skill progression [36].

## Thermal injury

The avulsion of small branches and side branches being cut very close to the vein causes more thermal injury during the initial learning curve period [24]. Many side branches are cut very short near the popliteal area due to superficial leg veins and patients with thin legs due to the dense fibrous tissues which lead to thermal injury on the vessel wall. Patients with abnormal leg anatomy need special attention as these cases are more complicated. This has to be taken into consideration during the learning curve period, which promotes excess thermal spread on the vein during coagulation of the difficult side branches near the knee area [37]. Patient selection for novice practitioners is an important consideration for avoiding exacerbated damage to the vein.

## CO_2_ insufflation

Additionally, it has been recently suggested that sustained CO_2_ usage during harvesting may promote an acidic local environment, negatively influencing endothelial integrity [26]. Inexperience and unfamiliarity with the endoscopic technique is associated with longer harvesting durations, resulting in prolonged exposure to CO_2_, which may contribute to damage to the intimal structure. Other studies also show that endothelial integrity may be compromised due to the effects of temperature, pH, distension and composition of storage solution and that endothelial integrity is superior with the no-touch technique [38]. Recent studies assessing the effect of pH on endothelial cell viability concluded that pH 7.3–7.4 is optimal for endothelial preservation, whereas more acidic environments are harmful [39]. Prolonged use of CO_2_ and conversion from EVH to the OVH technique causes greater damage to the vessel wall and are more prevalent during the learning curve period.

It is therefore pertinent to ask, is endoscopic vein harvesting only suitable for skilled harvesters, and not for junior practitioners? If so, how can we alter the current training practices in order to minimise the effect of practitioner inexperience on patient safety?

## Skills acquisition

Endoscopic vein harvesting is technically challenging and requires new psychomotor skills that differ from those needed in traditional open vein harvesting. Research into determining the best method of training new practitioners is sparse within the literature, despite growing evidence of increased conduit damage by inexperienced harvesters. Although a multitude of factors have been demonstrated to affect technical skill acquisition [40], there remains a paucity of surgical tools developed on the basis of the common problems encountered with the procedure.

## Training methods in other surgical specialties

The competency-based training curriculum for endoscopic surgery is available in other surgical specialities, although they also insist on valid tools, which enable the trainee to practice on a series of training activities [41]. A procedure based progressive surgical skills curriculum must begin by slowly introducing the basic skills necessary for endoscopic surgery. This is important so that learners improve their hand-eye coordination and become well versed on the procedure and equipment, the fulcrum effect and depth perception [42]. Procedural training enables integration of judgement and knowledge into the technical skills already learned [43], whilst the progressive structure of the learning process reduces the stress level of the trainee practitioner.

In laparoscopic surgery, training on inanimate video trainers and virtual reality simulators has been shown to improve the surgical performance on real patients [44]. However, structured competency-based surgical training tools do not exist for endoscopic vein harvesting and require validation in terms of which tasks should be performed, at which experience level, for how long and how often. Also, there is currently no standard set of benchmark criteria to allow progression [45].

A training tool needs to be designed to include criteria based on common problems experienced by novice EVH practitioners when using current training protocols. To be an effective surgical tool, the curriculum has to provide both theoretical and technical knowledge, and performance evaluation. It must also be meaningful and informative to the novice practitioner [46]. This is an extremely important issue, and requires in-depth evaluation of a new training programme, tailored specifically for EVH yet building upon expertise from other successful specialities.

## Patient selection criteria during EVH training

The selection of patients for endoscopic vein harvesting is important [47]. The risk involved in selecting patients with diabetes, peripheral vascular disease and abnormal or diseased veins might predispose to accelerated myointimal proliferation which leads to luminal narrowing and occlusion of the vein graft [48]. Additional damage caused by the novice harvester during training can accelerate the natural progression, leading to early occlusion and graft failure. Varicose veins are very thin walled veins with loss of elasticity, which are associated with a high risk of rupture compared to normal vessels [49]. The quality of the vein needs to be checked carefully as a major confounding factor, as not all patients are suitable for endoscopic vein harvesting. Vein quality can be assessed using pre-operative vein mapping [50]. During the learning curve period, there is an increased risk of small branch tear of the vein (this risk is significantly reduced with operator experience) [51]. Thin legged patients with superficial veins have more hair line branches with a high risk of tearing during endoscopic vein harvesting [20]. Therefore, it is logical that exclusion of these patients during the early stages of operator training could decrease risk of vessel damage, and consequently improve operator confidence. The gradual introduction of more complicated patients as the practitioner gains experience is advised in order to minimise the inherent risk in the procedure.

## Progressive learning

Adopting any new technique or technology adds an increased risk of injury to the patient, and there is a well reported learning curve period for EVH, which ranges from 5 to 30 cases [52]. Learning for prolonged periods and training under direct supervision is not always practical in the clinical setting. However, progressive learning using a structured curriculum can enable the trainee to alleviate their stress and anxiety during the learning curve period to reduce complications [53].

Recent evidence suggests that the progressive use of endoscopic techniques reduces operative time, operative complications and also alleviates the stress of the operator [32]. In addition it may be beneficial to limit the trainees to harvest one length of vein initially until they are proficient. The gradual introduction of two length and three length harvesting can allow operators to increase confidence and preserve vein integrity.

## Surgical skills

Surgical skills acquisition for endoscopic vein harvesting requires prior detailed knowledge of the anatomy of the leg. Problems arise during training as a result of inexperience in appropriate handling of endoscopic equipment [32], combined with inexperience of how to tackle difficult situations, such as double vein branches, bleeding inside the tunnel and obese patients. These aspects need to be included in the surgical curriculum prior to the operator harvesting independently in order to avoid any potential complications to the leg and vein.

## Identifying the vein and performing skin incision

The long saphenous vein (LSV) is still the most common conduit [1] for CABG surgery due to its long length, versatility and ease of access. Traditionally, the LSV is harvested via a skin incision performed near the medial malleolus along the medial aspect of the knee. This is a very simple place to identify the vein in most patients [54]. However for the EVH technique, the LSV skin incision is performed as a 2 cm transverse incision above or below the knee, or both, depending on the length of conduit required for the surgery [54].

Some studies have suggested that identifying the vein near the knee is very difficult for inexperienced or junior operators [1, 52] (as well as experienced practitioners in difficult patients [50]). The incorrect location of skin incision on the donor leg can promote the formation of flaps and difficulty finding veins, especially in obese patients [50, 54]. The current literature has reported that central lines in many centres are inserted with the guidance of ultrasound, which decreases complications and improves success rate [55, 56]. Ultrasound systems are easy to use, and require no novel training or equipment (this has been used in central line placement and other surgical procedures for a number of years). The use of pre-operative and intra-operative ultrasound systems can prevent complications associated with identifying the vein near the knee or thigh [50]. Appropriate training of the operator to identify the LSV and perform the appropriate skin incision is essential for EVH.

## Handling of equipment

The handling of endoscopic vein harvesting equipment is technically challenging and is also very complicated to use in a high-pressure, stressful environment. Detailed prior knowledge and practice of handling the equipment is very important, and is a stage in EVH skills training that can be monitored carefully, where improvements or corrections can be made prior to the procedure being performed on patients. Animal leg models are the only available tool for practicing EVH with endoscopic equipment. Although these models are useful as an early introduction to EVH, they are very expensive and do not represent an appropriate tool for long term training. Ideally a short training session including vein dissection and branch isolation should be performed, and certified with formal assessment by a senior practitioner.

Current literature suggests that poor graft patency and clinical outcome associated with novice EVH harvesters is due to a tendency to handle the equipment forcefully, thus manipulating and damaging the vein [23]. There is also histopathological evidence to suggest that vein stress is greater at the base of the vein branches [6]. This identifies inexperience and unfamiliarity with the appropriate method of handling the equipment. If the learner undergoes formal structured training, then this can be eliminated and graft stress can be minimised or prevented.

## Vein dissection

This is the initial step in the EVH process, and is crucial for preservation of the vein layer integrity during harvesting. The vein consists of three layers, with the outermost layer of the adventitia playing a vital role in preservation of the vasa vasorum [57]. Injury to the adventitia and stress on the vein can lead to intimal damage [58]. In open vein harvesting, using the “no touch technique” can preserve the adventitia and perivascular tissues, reducing graft remodelling [23, 58]. This technique also preserves functional, structural and mechanical features of the vein wall. It is suggested in the literature that practitioners should dissect the vein with surrounding fat during open vein harvesting to preserve the adventitia and perivascular tissues [21]. Our experience has shown that dissecting the vein with surrounding fat during EVH reduces the number of small avulsions, conferring long-term benefit on graft patency (unpublished data). Skeletonising of the vein and trauma to the vessel wall causes endothelial disruption, which leads to diminished production of nitric oxide and smooth muscle cell proliferation. This is a primary contributing factor for intimal hyperplasia [38, 59].

## Nitric Oxide (NO) production and smooth cell proliferation

**Fig. 1:**
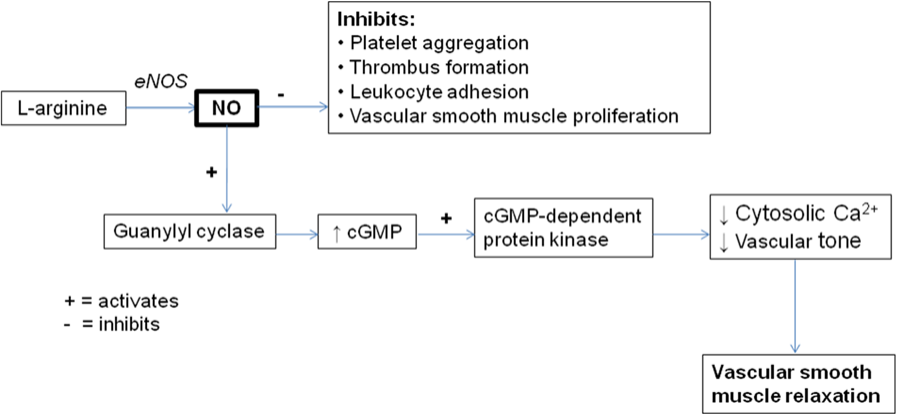
The effects of nitric oxide. Nitric oxide (NO) is an important vasodilator synthesised from L-arginine via endothelial nitrogen oxide synthase (eNOS). NO activates guanylyl cyclise, leading to elevated concentrations of cyclic guanosine monophosphate (cGMP) and the downstream activation of cGMP-dependent protein kinase. This enzyme mediates a significant reduction in intracellular Ca^2+^concentration and decreased vascular tone, resulting in vascular smooth muscle relaxation. Simultaneously, NO inhibits the aggregation of platelets, vascular smooth muscle cell proliferation, thrombus formation and ameliorates the adhesion capabilities of leukocytes

In addition to its role as a physical barrier, the endothelium is of great importance with protective features including anti-thrombotic, anti-spasmodic and anti-atherosclerotic activities, (Fig. 1). The most important endogenous vasodilator is nitric oxide (NO), a potent endothelium-dependent vasorelaxant synthesised from the amino acid L-arginine by endothelial nitric oxide synthase (eNOS) [60, 61]. When this protective mechanism is absent or rendered ineffectual, adverse clinical outcomes may ensue, thus explaining some of the conflicting findings in recent clinical trials [62, 63]. Endothelial denudation has been proven to be detrimental to veins as it decreases functional capacity and augments the likelihood of thrombosis. The learning curve period is associated with greater damage during vein dissection and branch isolation, which impacts on conduit quality.

## Branch isolation

This is the final stage of the EVH technique, and imparts more pressure on the vein branch which leads to intraluminal tears at the base. Several reports support the notion that this type of tear occurs more frequently within experienced operators [1, 6]. The protection of nitric oxide and the secretion of prostacyclins and matrix occurs immediately due to vein stress, which leads to early positive remodelling of the LSV [23, 64, 65]. The major stress on the base of the branch during harvesting causes intimal injury which leads to platelet adherence, release of mitogenic proteins, smooth muscle cell proliferation and intimal hyperplasia [18, 66, 67]. Therefore, protection of the vein branch is essential. It is crucial to learn this part of the technique progressively under proper supervision as the risk of complications is reduced when trained in this manner [53].

## Requirement for a validated vein scoring system

There is currently no standard validated vein quality assessment tool in cardiac surgery. There is a substantial degree of inter-patient variation in vein calibre and anatomical quality. However, understanding this and assessing the vein for damage accrued during harvesting plays a crucial role. A variety of categorical (i.e. ‘poor’, ‘fair’ and ‘good’) and numerical scoring systems have been formulated to assess harvested veins [68, 69]. An effective scoring system should aid the surgeon in determining the suitability of the conduit for grafting, and as such cover several criteria. In order to assess harvesting injury, parameters such as number of vein repairs including small avulsions, bruising to the vein, the size of side branches and the calibre of the vein must be taken into account. There are a significant number of additional vein repairs required and substantial bruising observed during the learning curve period due to increased traction applied to the vein [70]. Currently, physical examination by the operating surgeon is the standard practice in determining whether a conduit is acceptable for use in bypass surgery, and as such there is significant variation in the quality of grafts utilised. This important area needs to be explored and this discrepancy addressed in order to standardise patient care. There is therefore an urgent requirement for a validated vein scoring system for minimally invasive vein harvesting techniques.

## Economic burden

Vein graft failure or occlusion is one of the major reasons for patient readmission for coronary stenting or redo CABG surgery [71]. It imparts a huge economic burden on resources and is a stressful situation for the patient. Current evidence suggests that 30–40 % of CABG patients require re-interventions within a decade of their first CABG surgery [72–74]. Repeat interventions carry a significant risk and increase the cost of healthcare. However, an economic analysis study reported that EVH is the most cost-effective method because of the short term benefits such as lower incidence of wound related complications and early hospital discharge, when compared to open techniques [10, 54, 75]. The provision of didactic and vocational training to surgical operators may reduce economic burden further, by reducing complications associated with inexperience.

## In-house training

Recent evidence contests these findings, concluding that EVH is not a cost-effective method within the first 35 days, compared to open harvesting [76]. Yet, this paper included the cost of learning the procedure, inclusive of external courses and training cases, totalling $10,000. The development of a structured, validated learning tool that can be effectively taught in-house should minimise the costs associated with training novice harvesters.

## Cost analysis

The cost of training the novice practitioners with the structured training will be more expensive than current standard training over the short term; however this should be compensated by improved conduit quality and better long term outcome. A recent meta-analysis demonstrates that the length of hospital stay is significantly reduced (95 % CI – 1.08 to 0.12) in EVH compared to open vein harvesting which includes 4522 patients from seven RCT and eight observational studies [22].

## Structured training

Our recent experience with the utilisation of a structured training programme highlights the potential benefits of this training pathway over current standard training procedures. Significant improvements in vein conduit quality were demonstrated alongside increased operator confidence when EVH was performed by practitioners trained using the Manchester Endoscopic Learning Tool (MELT) programme [77]. Many centres have now adopted this structured training pathway as standard EVH training.

## Discussion

The volume of EVH procedures performed for CABG surgery is increasing steadily [6]. As such, there is a requirement for the practitioners to be trained with the structured training programme to obtain high quality vein conduits. Meticulous preservation of layers of saphenous vein during harvesting is an important factor in determining the graft patency rate [78]. The integrity of the vein is affected by many factors; however, preventing additional avoidable damage during EVH training is essential. This can be achieved by using a surgical skill curriculum training tool, which should be structured, reliable and rigorously validated to be incorporated into an objective clinical assessment. This will analyse an individual’s development and allow progression through a structured training programme [79]. This programme should contain elements that are specifically designed to minimise the likelihood of complications that are commonly observed during the learning curve. This includes theoretical knowledge to reinforce the importance of preserving the integrity of the vein, equipment training to improve hand-eye co-ordination and a gradual introduction to clinical practical skills to eliminate vein damage. The training programme should be evaluated on every step using a validated Objective Structured Assessment of Technical Skills (OSATS) which have been proven to have reliability and construct validity in measuring general operative minimally invasive surgical skills that are applicable to all surgical procedures [80].

## Conclusions

In order to combine the didactic and clinical skills training for novice EVH learners, it is necessary to promote a change in culture locally, but also and more importantly within the wider cardiothoracic community. The whole process could be time consuming but it is an essential element of obtaining a good quality vein conduit for a better clinical outcomes.
